# 

**Published:** 1900-04-21

**Authors:** 


					yffitlfoTriTALr " "The standard of highest purity/'?Lancet.^: ~ ; J April 21st, 1900,
CADBURrs < H 1? 0AR?"RY'S
COOOA
COCOA
NO DKUOS OR AL.KAL.IK8 U8KO.
rO,Vnmn?nn Tormo l    ?? ?'
No. 70or Vol. XXVIII. Saturday,
April 21, 1900.
["Subscription Terms,^ pRICE TWOPENCE.

				

